# Fees for Professional Services

**Published:** 1860-02

**Authors:** 


### FEES FOR PROFESSIONAL SERVICES.

FROM THE NORTH AMER. MEDICO-CHIRURGICAL REVIEW.

The remunera.ion for medical, surgical and obstetrical services have, of necessity, varied very much in different ages and countries, according to the estimate of the services rendered, or the relative value of money. In many countries, towns and cities, there are regular fee-bills regulating the charges for ordinary practice; a custom which we have always regarded as absurd and unjust, since it places, in this particular, all practitioners, whatever may be their respective merits, upon the same level, whereas every man should be permitted to charge according to his skill and the nature of his services, not forgetting the circumstances of his patients.

In looking, not long ago, over some of our papers, our eye chanced to light upon a bill for professional services rendered by the late Dr. James Craik to Captain G. S., of Washington City, the father of a large and highly respectable family, to one of the members of which we are indebted for this interesting document. Dr. Craik was the physician of Washington and attended him in his last illness, in conjunction with Dr. Dick. The bill is dated 1795, beginning in April of that year, and ending in March, 1799, the entire amount being £66 16s, 6d. It covers more than ten pages of foolscap, aud particularizes every item with the same care as a merchant's or grocer's account. The following extract will serve as a specimen of Dr. Craik's charges:—

| | £ | s. | d. |
|---|---|---|---|
| For extracting Peter's tooth,........................ | 0 | 3 | 0 |
| Visit to your lady, and anodyne draught,.......... | 0 | 3 | 0 |
| A purge,.................................................. | 0 | 1 | 3 |
| An emetic,............................................... | 0 | 1 | 6 |
| For bleeding Capt. S.,............................... | 0 | 3 | 0 |
| " delivering your lady,............................ | 5 | 0 | 0 |
| Visit to your son George, and vermifuge pill,...... | 0 | 1 | 0 |
| 23 syphilitic pills for Sam.......................... | 0 | 16 | 0 |
| Visit to your child Harriet, and 2 alterative powders, | 0 | 5 | 0 |
| Visit to your son George, and vermifuge pill,...... | 0 | 3 | 0 |
| 8 ounces of injection for boy Sam,................. | 0 | 5 | 0 |
| One syringe,............................................ | 0 | 3 | 0 |
| Dressing negro boy's hands,......................... | 0 | 2 | 6 |
| Visit to your lady, and anti-rheumatic tincture,..... | 0 | 3 | 0 |
| Visit to your lady, and opening abcess in breast,.... | 0 | 5 | 0 |
| Blistering plaster,..................................... | 0 | 5 | 0 |
| Bleeding,................................................ | 0 | 3 | 0 |
| 12 febrifuge powders,.................................. | 0 | 6 | 0 |
| 6 ounces best olive oil,............................... | 0 | 2 | 6 |
| Bleeding negro woman,............................... | 0 | 3 | 0 |
| " Master George,................................ | 0 | 3 | 0 |
| Dose of salts,........................................... | 0 | 1 | 6 |
| Inoculating your child,................................ | 1 | 0 | 0 |

Emetics, purgatives, absorbent powders, anodyne draughts, and preparations of bark—powder and infusion—are prominent items of the account.

The custom of presenting items in medical bills, has, we presume, become obsolete. It is certainly inconsistent with dignity of a professional man of the present day to descend to such minutiæ. A round statement in dollars should be quite sufficient. Only once, in our whole life, have we been requested so to demean ourselves. We replied, that it was contrary to the habits of professional gentlemen to specify their charges, with the minute exactness of an auctioneer's catalogue, at the same time that we informed our *patron* our ledger was at his service; nay, furthermore, that if in future he wanted our attendance on such conditions, he could not have it. The *creature*, now one of the merchant princes of the city where we then resided, had all his life been dealing in sugar, molasses, and whisky, and could therefore not help following the force of his habits.

Professional services are generally much more highly appreciated in cities and large towns than in the country. In the United States, practitioners are much better rewarded in the South and Southwest than in the North and East; in the slave States universally much more liberally than in the free. The reasons for these practices are obvious. In towns and cities, physicians could not live if their charges were not higher than they are in the country; and in the Southern regions of the United States, money is much more plenty than among the same number of inhabitants in the North and East.

The highest fees for medical services in this country, are paid at New Orleans, where the ordinary charge for a visit is from two to five dollars, while consultation services yield at the rate of from ten to twenty. At Charleston the first consultation fee, established by long habit, is $14,00, the subsequents ones being each $2,00. In this city the first consultation visit is usually $5,00, and those made afterwards, $2,00 each. The fees of surgeons are generally, everywhere, higher than those of physicians.

Since our attention has been directed to this subject, we have examined a number of works in our library with a view of ascertaining the charges, ordinary and extraordinary, of practioners, dead and living, in different countries and in different ages.

In ancient times some remarkable fees were obtained for professional services. It is related of Charmis, who kept a bathing establishment at Rome, in the reign of the Emperor Claudius, that his regular charge for advice to those who were anxious to avail themselves of his treatment, was £800. He was the first water-cure doctor, if we may credit the researches of Dr. Doran, that ever practiced, and he made an immense fortune, such as no brother of the craft of the present day can at all approach.

The most liberal fee of modern times was that received by Dr. Dinsdale, a physician of Hertford, England, for inoculating the Empress Catherine, at whose request he visited Russia in 1768. The operation was perfectly successful, and such was the gratification of the Empress, that she made Dinsdale a baron of the empire, besides presenting him £12,000, and a pension of £500 a year.

The largest fee ever received by Sir Astley Cooper was 1000 guineas. His patient was a man of the name of Hyatt, a retired West India merchant, who was affected with stone in the bladder. The manner in which the fee was presented, is worthy of notice. When Hyatt had entirely recovered from the effects of the operation, he requested his surgeon, with his two medical attendants, Dr. Lettsom and Dr. Nelson, to visit him on a particular day. Cooper arrived after the physicians had left the room; he met them down stairs, discussing the liberality of their patient, who had presented each with £300. Sir Astley was cordially received by the old West Indian, and after having chatted a little while, he rose to take his leave, and had got as far as the door, when Hyatt threw his night-cap at him, saying at the same time, "There, young man, put that into your pocket." Upon examining it, he found a check in it for 1000 guineas.

Hyatt, it would seem, was equally as liberal to his apothecary, or regular family attendant. One day being sent for in haste to visit his patient, he fell down and hurt his knee. so as to cause him on entering, to be lame. Hyatt observing his condition, immediately exclaimed, "Dobson, old fellow, what is the matter?" On learning what the trouble was, he pulled out a £100 bank note, and applied to the joint, adding that it was the best plaster in the world for a bruised knee.

A wealthy London merchant, Mr. William Cole, paid Sir Astley Cooper annually, for years, £600, for attendance upon his family.

During the hey-day of his professional life, Sir Astley Cooper frequently made 100,000 dollars a year by his practice. Much of this vast sum was received for chamber practice.—He had to answer many letters of advice, for which he never received less than a one pound note, while many yielded him five times that amount.

Dr. Lettsom, who was a West Indian by birth, made, in a visit which he paid to Tortala, his native town, soon after having completed his studies in London, nearly £2000 in five months. After he had succeeded in establishing himself in the British metropolis, his income annually, ranged from twenty to twenty-five thousand dollars. In 1800, he received in fees, £12,000, or sixty thousand dollars.

Fothergill, the Quaker doctor, did an immense practice.—For the last twenty-five years of his life, his fees annually averaged nearly seven thousand pounds, or about thirty-five thousand dollars. He commenced his practice in 1740.

Mead's income was, on an average, from five to seven thousand pounds, for many years. He once received three hundred guineas for visiting a patient at Ingestree, in Staffordshire. The patient had been very ill, but recovered before the arrival of his great physician.

Dupuytren's income was enormous; he began life as a poor boy, and died worth more than a million of dollars.

Graefe, the celebrated surgeon of Berlin, left an immense fortune, the result of his professional labors.

In this country, physicians are not noted for their high charges or great income. One of the largest fees ever received by any one, was that of Dr. Ephraim McDowell, for the operation of ovariotomy, performed upon a lady in Tennessee, whose husband gave him fifteen hundred dollars. We have heard of a fee of five thousand dollars being paid to a New York surgeon for an operation of club-foot, but we are unable to vouch for the authenticity of the story. Physick left a large fortune, but rather in consequence of the rise in his estate than of his large professional emoluments. His charges were generally small. A gentleman once handed him a hundred dollar note, for attendance on his wife; but the doctor thinking that it was out of all proportion to the value of his services, returned all but ten dollars.

The salaries of court physicians and surgeons, have also varied according to the times in which they have flourished, and the respective ranks which they occupied. In the reign of Henry III, of France, the pay of the royal household staff was as follows:

*First Surgeon.*

Ambrose Pare,................666 livres and 12 sols.

*Surgeons in ordinary.*

Pierre Pegray,....................333 livres and 6 sols.
Antoine Portail,.................333 " " 6 "

*Assistant Surgeons, serving each three months in the year.*

January, February, and March.

Jacques Guillemean,......................100 livres.
Isaac Bruns,......................................100 livres.

April, May, and June.

Jehau Lambert,.............................100 livres.
Jacques D'Amboise,......................100 livres.

July, August, and September.

| | |
|---|---|
| Ismael Lambert,........................... | 100 livres. |
| Hierome de la Noue,....................... | 100 livres. |

October, November, and December.

| | |
|---|---|
| Charles Buchalier,......................... | 100 livres. |
| Michael Vandelon,.......................... | 100 livres. |

Louis XIV seems to have had a high appreciation of the services of his professional attendants. Being affected with anal fistule, an operation became necessary, on recovering from which he exhibited his gratitude by bestowing upon them not less than £14,700 in the following ratio:

| | | | |
|---|---|---|---|
| To M. Felix,.................. | 50,000 crowns, | or | £6000. |
| Dr. Duquin,................ | 100,000 livres, | " | 4000. |
| Dr. Fagon,................. | 24,000 " | " | 1000. |
| M. Bessiere,............... | 40,000 " | " | 1500. |
| Four apothecaries, each,..... | 3,000 " | " | 2000. |
| M. Raye, apprentice to M. Felix | 400 pistoles | " | 200. |

Considering the enormous price paid for the operation, it is surprising that the filthy disease which it was designed to relieve, should ever have become the fashionable court complaint. A surgeon of the present day may regard himself as extremely fortunate if he can occasionally get a patient who is able to pay him two hundred dollars, for the division of the sphincter muscles, including the after-treatment.

Scanzoni, professor of midwifery in the University of Wurzburg, received twenty-five thousand dollars for attending a short time ago, the Empress of Russia in her confinement. The prestige with which the favorable reception of this physician at the Russian court invested him, has rendered him the most celebrated accoucheur of continental Europe, and laid the foundation of one of the most aristocratic practices in the world, crowds of the German and foreign nobility flocking to him from all parts.

Medical men sometimes receive, in addition to their regular fees, large presents, either in money, plate, clothing, or wine. Thus, Ambrose Pare, the father of French surgery, at the siege of Metz, in 1552, had a tun of wine sent to him for curing one of the officers of a broken limb, by De la Roch, with a promise that "when it was dronken he would send mee another." The Sultan recently, after his recovery from an attack of ague, in which he was obliged to take an unusual quantity of quinine, the effects of which occasioned symptoms which somewhat alarmed the court, presented his physician,

Dr. Caratheodory, precious stones, works of art, and various other articles, valued at between twelve and sixteen thousand pounds, besides a handsome estate.

Physicians, on retiring from practice, are sometimes presented with a service of plate, by their grateful patrons; and similar compliments are occasionally paid by towns, and cities, in consideration of the services rendered by practitioners during the prevalence of devastating epidemics.

Sometimes, again, the present is in the form of a wife.—Thus, Podrilius, whose praises have been sung by Homer, was rewarded by the King of Caria with the hand of his daughter, whose life he was supposed to have saved by bleeding her in both arms, after a fall from the the top of a house. Such a gift might not always be agreeable or convenient to the recipient, but it could hardly be otherwise, when it comes in the form of a rich princess, as in the case of Podrilius.

Governments do not always reward their subjects in proportion to the value of their services. Jenner, for his immortal labors in vaccination, by which millions of lives have been preserved, received from the British Parliament the paltry sum of twenty thousand pounds.Brossar d, a French surgeon, in the seventeenth century, was richly rewarded by the French government for the disclosure that agaric would arrest hæmorrahge after surgical operations. The remedy was tried, and of course, found useless, though not till a number of lives had been lost by it. Mrs. Stephens, as late as the last century, obtained a large sum from the British Parliament for making known the supposed virtues of Castile soap and egg shells in urinary calculi.

The charges for attendance at coroner's inquests are not commensurate with the services exacted upon these occasions. From ten to twenty dollars is the usual fee for making a dissection for the benefit of the public, and even that sum is often grudgingly allowed. In cases of poisoning the remuneration is, of course, more liberal, though seldom adequate. The largest compensation for services of this description, ever paid in this, or, perhaps, in any other country, was that recently awarded by the city of New York to Dr. Doremus, Professor of Chemistry in the New York Medical College. The sum allowed to him was three thousand dollars, besides three hundred dollars for the outlay of new apparatus. The case was that of Stephens, tried for the murder of his wife by poison. Dr. Doremus was obliged to analyze two entire bodies.

Finally, a good fee is a powerful stimulant, causing the most delightful sensations, and goading a man on to the most vigorous performance of his duties. It increases the pace of the sluggard, and improves the digestion of the dyspeptic.—There is not a man in the profession, that has not, at times, felt the force of the practice of the celebrated physician of Bath. Finding himself no better for his own prescriptions, he laughingly observed to a friend, "Come, I think I will give myself a fee, I am sure I shall do better then." Putting his hand into his pocket, he took out a guinea, and gravely passed into the other. He soon got well. Assuredly, reader, there is great potency in a good fee.

Physicians sometimes place no better estimate upon their services, than their patients. A young professional acquaintance recently told us, that not long ago, after having prescribed for a female, she handed him a one dollar counterfeit note, which he did not hesitate to take, although he knew at the time that it was worthless, believing that it was a fair equivalent for his services. We have not examined our brother's organ of conscientiousness, but suppose it to be very large.

